# Antibody Recognition of Highly and Low-Pathogenic A/H5Nx Influenza Viruses in Sera of Mexican Donors

**DOI:** 10.3390/pathogens15040352

**Published:** 2026-03-26

**Authors:** Maritza Cordero-Ortiz, Mario Solís-Hernández, Marlen Cayetano-Mondragón, Nadia Carrillo Guzmán, Olivia Valenzuela, Verónica Mata-Haro, Luis G. Giménez-Lirola, Jesús Hernández

**Affiliations:** 1 Laboratorio de Inmunología, Centro de Investigación en Alimentación y Desarrollo, Hermosillo 83148, Sonora, Mexico; maritzacoordero@gmail.com; 2 Comisión México-Estados Unidos para la Prevención de la Fiebre Aftosa y otras Enfermedades Exóticas de los Animales (CPA), Servicio Nacional de Sanidad, Inocuidad y Calidad Agroalimentaria (SENASICA), Secretaría de Agricultura y Desarrollo Rural (SADER), Ciudad de México 05110, Mexico; mario.solis@senasica.gob.mx (M.S.-H.); dgsa.iica103@senasica.gob.mx (M.C.-M.); nadia.carrillo@senasica.gob.mx (N.C.G.); 3 Departamento de Ciencias Químico Biológicas, División de Ciencias de la Salud, Universidad de Sonora, Hermosillo 83000, Sonora, Mexico; olivia.valenzuela@unison.mx; 4 Laboratorio de Microbiología e Inmunología, Centro de Investigación en Alimentación y Desarrollo, Hermosillo 83304, Sonora, Mexico; vmata@ciad.mx; 5 College of Veterinary Medicine, Iowa State University, Ames, IA 50011, USA; luisggl@iastate.edu

**Keywords:** H5N1, H5N2, pandemic, cross-reaction, hemagglutination inhibition test

## Abstract

Influenza A viruses (IAVs) are among the most common sources of new pandemic strains in humans. Spillover from birds to mammals can lead to viral adaptation in new hosts, as seen with IAV H5N1. H5N2 viruses have also been transmitted successfully to humans through contact with infected birds and poultry. In the present study, we evaluated the presence of antibodies against H5Nx viruses in serum samples from a Mexican adult population (*n* = 476) using the hemagglutination inhibition (HI) test. The analysis included comparisons between sex and age groups. Potential epitopes recognized in the H5 proteins of each strain were predicted using Ellipro. We detected antibodies against H5N1 in 2.5% of the samples, with the highest HI titers of 1:64. The proportion of positive samples for H5N2 2006 was higher (18.9%) than for H5N2 2024 (10.7%). The oldest groups (>50 years old) showed the highest proportion of positives for both viruses, whereas the youngest was for H5N1. These results demonstrate a low proportion of cross-reactive antibodies against the IAV H5N1.

## 1. Introduction

Influenza A viruses (IAV) belong to the *Orthomyxoviridae* family and the *Alphainfluenzavirus* genus [1,2]. IAV is a seasonal pathogen with pandemic potential, and its primary reservoir is wild birds. IAVs can be classified as highly pathogenic or low-pathogenic avian viruses. H5N1 virus clade 2.3.4.4b is a highly pathogenic avian virus that has been transmitted through wild birds to several countries worldwide and is considered panzootic, with numerous cases in wild birds, poultry, and mammals since 2022 [3,4]. The adaptation of this virus to mammals is a concern, especially given its adaptation to dairy cows [5]. In Mexico, clade 2.3.4.4b was identified in wild birds for the first time in October 2022 [4]. Besides H5N1, H5Nx viruses include H5N2 viruses, which are also associated with infection of wild birds but are rarely transmitted to humans [6,7]. The first fatal human infection with low-pathogenic avian influenza H5N2 was reported in Mexico on 23 May 2024 in a 59-year-old resident of the State of Mexico, who was hospitalized in Mexico City and died despite having no known exposure to poultry or other animals [8,9]. Health authorities confirmed that a case notified on 30 September 2025—initially classified as avian IAV (H5)—corresponded to avian IAV (H5N2), representing the country’s second documented human infection with this subtype [10].

Hemagglutinin proteins are highly similar among IAV subtypes, avian or human, and can share epitopes recognized by the host’s immune system. Humans typically become infected with H1N1 and H3N2 subtypes seasonally [11]. Due to constant exposure to these viruses and annual vaccination, these infections are self-limiting in the main part of the population [12]. Some studies have shown that antibodies against the seasonal influenza virus can protect against H5N1 infection [13]. Additionally, the presence of H5N1 antibodies in humans may be due to immune imprinting [14]. The presence of antibodies in humans can help assess the extent to which they are prepared to respond to potential infection with H5N1 or H5N2 IAVs. In this study, the presence of antibodies against three IAVs (H5N1, H5N2 2006, and H5N2 2024) was evaluated in serum samples from Mexican blood donors, and potential epitopes were predicted for each H5Nx hemagglutinin.

## 2. Materials and Methods

### 2.1. Serum Samples

We evaluated human serum samples from 139 donors and 45 plasma receivers, as well as sera (*n* = 292) collected at the beginning of 2022, as part of previous COVID-19 studies [15,16]. The Ethics Committee of CIAD evaluated and approved this study (CEI/005-2/2020), and all participants provided informed consent. Demographic data included 262 females and 214 males aged 18 to 65 years, classified into five age groups (Table 1).

### 2.2. Hemagglutination Inhibition (HI) Test

The presence of antibodies against H5Nx influenza viruses was evaluated by the hemagglutination inhibition test. Three different viral strains were included in the analysis: H5N2 strain A/Gallus gallus/texcoco/CPA-01654-24/2024 (GenBank ID: XBU81388.1); H5N1 strain A/Falco_rusticolus/EdoMex/CPA-19638-22/2022 (GenBank ID: UYL41378.1); and H5N2 strain A/chicken/Durango/2393-06/2006(H5N2) (GenBank ID: AIN25336.1).

The HI test was performed as previously described [17]. Complete viral inactivation was confirmed by two successive blind passages in specific pathogen-free (SPF) embryonated chicken eggs, with no embryo mortality and no hemagglutinating activity. Viral identity was verified by subtype-specific qRT-PCR targeting the H5, H7, N1, N2, N3, and APMV1 genes (Supplementary Table S1) and by the corresponding serological assays required for subtype confirmation. Hemagglutination titers were determined using 1% chicken red blood cells and were expressed as log_2_ values. All antigen preparations were aliquoted, labeled with virus strain, passage number, inactivation date, and hemagglutination units (HAU) titer, and stored at −80 °C until use.

To ensure complete removal of non-specific hemagglutination inhibitors and improve the accuracy of antibody detection, all serum samples were processed using the trypsin–heat–periodate (THP) treatment protocol as described in the WHO Manual on Animal Influenza Diagnosis and Surveillance (Annex F, Alternate Protocol II) [18]. Briefly, sera were treated with trypsin, heat-inactivated, and subsequently oxidized with potassium periodate prior to hemagglutination inhibition (HI) testing. This protocol provides an efficient method for eliminating non-specific inhibitors present in mammalian sera and is recommended for the reliable assessment of antibodies against potentially zoonotic avian influenza viruses, including H5 and H7 subtypes. Additionally, kaolin and red blood cells were added to remove hemagglutination inhibitors. After this, the HI test was performed by making double serial dilutions of the sera and mixing with four hemagglutination units of each virus. Then, these dilutions were incubated at room temperature (21 °C) for 30 min. After this, 1% avian erythrocytes were added and incubated for an additional 30 min at room temperature. Samples were considered positive if HI titers were 1:32 or greater.

### 2.3. Viral Propagation

Virus propagation was performed in 9–11-day-old specific-antibody-negative (SAN) eggs according to standard WOAH guidelines. Briefly, clarified clinical samples and previously isolated viral stocks were inoculated into the allantoic sac using aseptic technique and incubated at 37 °C with 55–60% relative humidity for 48–72 h. Eggs were candled daily to monitor embryo viability. After incubation, allantoic fluids were harvested under sterile conditions and tested for hemagglutinating activity using 1% chicken red blood cells [19]. Hemagglutination-positive fluids were aliquoted and stored at −80 °C until further molecular and antigenic analyses. All manipulations involving highly pathogenic avian influenza viruses were carried out in a type A2 biological safety cabinet within biosafety level 3 (BSL-3) facilities, in accordance with institutional biosecurity, biosafety, and bioprotection standards.

The viruses were inactivated with β-propiolactone (BPL) according to the manufacturer’s recommendations. Residual BPL was hydrolyzed by incubation at 37 °C for 2 h, and the inactivated viral suspension was adjusted with 10% (*v*/*v*) phosphate-buffered saline (PBS, 0.01 M, 10X).

### 2.4. In Silico Analysis of the H5 Proteins

Sequences of the hemagglutinin from the three viruses were aligned using MEGA11 and visualized in Jalview 2.11.4.0 (H5N2 2024, GenBank ID: XBU81388.1; H5N1 2022, GenBank ID: UYL41378.1; and H5N2 2006, GenBank ID: AIN25336.1). H5 protein structures from the three strains were modelled using ColabFold v1.6.1: AlphaFold2 using MMseqs2, and the resulting structures were analyzed in Ellipro using the Bepipred Linear Epitope Prediction tool to identify possible linear and conformational B cell epitopes, considering a threshold of 0.5. Pairwise alignment of the sequences from the predicted epitopes was used to determine the similarity percentages between strains.

### 2.5. Statistical Analysis

The differences in antibody titers among strains were compared using the Kruskal–Wallis test. A Z test for proportions was performed to compare the proportions of positive samples between males and females, and a chi-square test for trend was used to evaluate this association across five age groups. Spearman’s rank correlation test was performed between the three different strains. All analyses were performed in GraphPad Prism version 10.

## 3. Results

### 3.1. Serological Results for H5Nx Hemagglutination Inhibition Test

**Figure 1:**
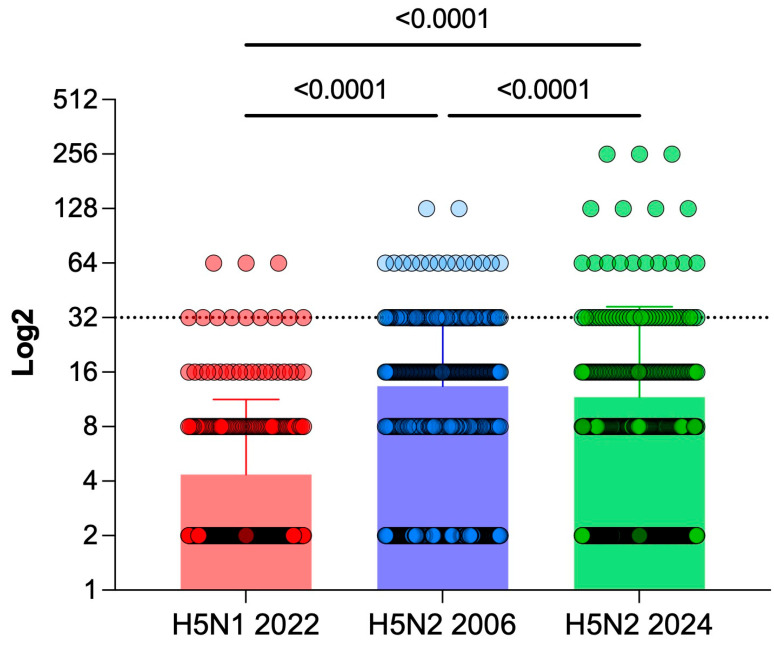
Antibodies against H5N1 and H5N2 strains in human samples. H5N1 2022 strain is shown in red, H5N2 2006 strain is shown in blue, and H5N2 2024 strain is shown in green. Each dot represents one sample. The X axis shows the titer at which the sample was able to inhibit hemagglutination for each strain shown on the Y axis. Titers ≥ 1:32 were considered positive, and the dotted line represents the cut-off. The bars represent the mean for each viral strain, and the line above represents the standard deviation.

The presence of antibodies capable of inhibiting hemagglutination for three viral strains expressing the H5 protein was evaluated. The highest titers were observed against the H5N2 2024 virus (up to 1:256), and this strain was the second most common among positive samples (10.7%; 51 of 476). The H5N2 2006 virus showed more positive samples (18.9%; 90 of 476) with a maximum titer of 1:128. The antibodies against the H5N1 strain had the lowest titers (1:64), and the lowest positivity in samples (2.5%; 12 of 476) among strains (Figure 1). There was a significant difference in antibody levels against H5N1 compared with H5N2 in 2006 and 2024 (*p* < 0.0001 in both comparisons). There was also a significant difference between the anti-H5N2 2006 and H5N2 2024 antibodies (Z = 5, *p* < 0.05).

**Figure 2:**
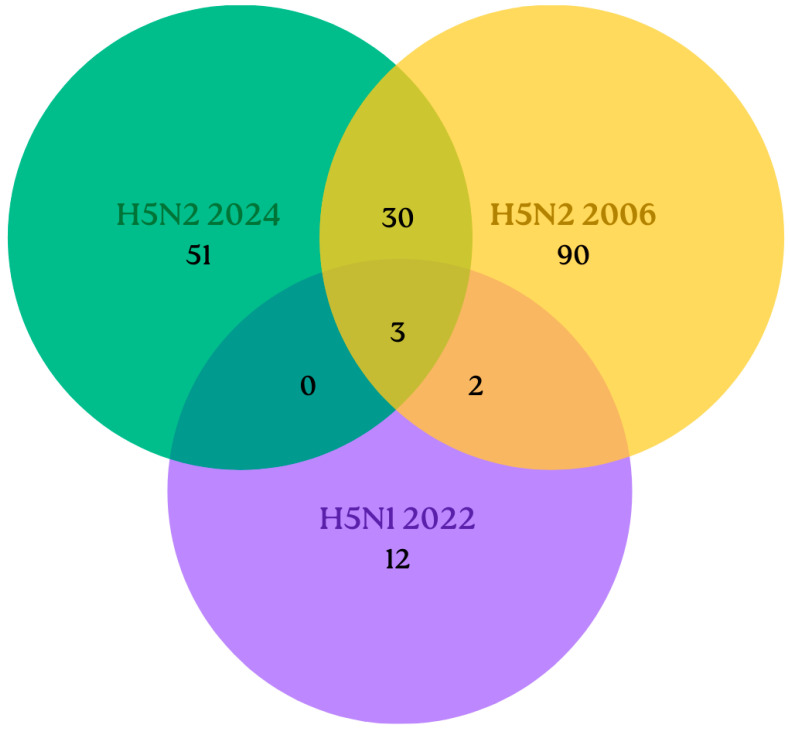
Venn diagram for positive human samples in the hemagglutination inhibition test for each viral strain evaluated, considering a threshold of 1:32 for the HI test. Each viral strain is shown in a different color: green for H5N2 2024, yellow for H5N2 2006, and purple for H5N1 2022.

Paired results of all the serum samples showed a higher correlation between H5N2 2024 and H5N2 2006 (r = 0.59; *p* < 0.001), but lower between H5N1 and H5N2 2006 (r = 0.291; *p* < 0.001) or H5N1 and H5N2 2024 (r = 0.265, *p* < 0.001). Accordingly, some positive samples recognized more than one viral strain, likely due to cross-reactivity. Only 3 samples were positive for the three viruses (0.6%). A total of 30 samples recognized both H5N2 viruses (6.3%), while only 2 samples recognized H5N1 and the H5N2 2006 strain (0.4%), and no samples recognized both the H5N1 and H5N2 2024 strains (Figure 2).

### 3.2. Age and Sex Association with Antibodies Against H5Nx Viruses

The results according to age and sex (Table 1) showed an association of antibodies for H5N2 2024 and age (*p* < 0.05), where positivity tended to be higher in the ≥61 years old group (20%; 2 of 10), followed by the 51 to 60 years old group, and positivity tended to decrease with age. Also, the male population was slightly more positive than the female population; however, this difference was not statistically significant (12.6% vs. 9.2%). The female population and the youngest age group had the highest titers (1:256) against the H5N2 2024 strain, although the youngest age group had the lowest positivity rate.

Similarly to the H5N2 2024 virus, antibodies against the H5N2 2006 virus showed an association with age (*p* < 0.05), with increased positivity in the ≥61 age group (70%; 7 of 10), suggesting a tendency for a decrease in the younger population. According to sex, the male group had more positive samples than the female group (22.4% vs. 16%; *p* < 0.05). In this case, both groups had the same highest titers (1:128). Additionally, the highest titers were observed in the youngest and the 41–50-year-old group.

In the case of antibodies against H5N1, there was no significant association between age and presence of antibodies (*p* < 0.05). However, the youngest group, aged 18 to 30 years old, had a higher proportion of positive samples (6.3%; 5 of 79), compared to the other groups. There was also slightly more positivity in the female group compared with the male group, but this difference was not significant (3% vs. 1.9%, *p* > 0.05), and the highest titers for both females and males were the same. The three youngest groups exhibited the highest titers of inhibition, decreasing as age increased.

### 3.3. Predicted Epitopes Recognized by Antibodies in Adults’ Sera

**Figure 3:**
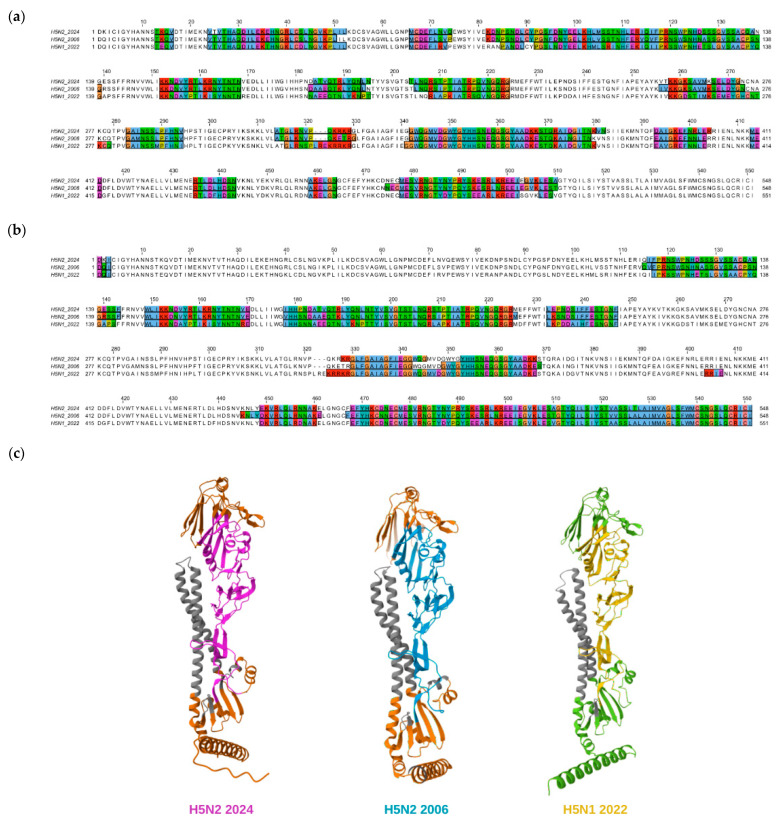
Linear and conformational epitope predictions for H5 proteins of three different H5Nx viruses. (**a**) Alignment between hemagglutinin protein sequences, amino acids related to linear epitopes, and (**b**) conformational epitopes are highlighted in color. (**c**) Predicted structural representations of each hemagglutinin HA1 region are shown in different colors: pink for H5N2 2024, blue for H5N2 2006, and yellow for H5N1 2022. Conformational epitopes of the H5N2 strains are shown in orange, and the H5N1 strain is shown in green. The HA2 region is shown in grey for all proteins.

Antibodies can recognize both linear and conformational epitopes. To determine the potential common sites recognized by positive samples, we sought to model and predict them. Conformational and linear epitopes were predicted for the three H5 proteins using Ellipro and subsequently aligned across proteins to determine the similarity percentage (Supplementary Tables S2 and S3). The three proteins had linear epitopes in very similar positions, with approximately 16 predicted epitopes (Figure 3a). However, 12 epitopes shared a higher similarity percentage between H5N2 strains than with H5N1, which could explain why more samples recognized both H5N2 strains. Two epitopes showed 100% similarity across all proteins, which could be those recognized by the 3 samples that inhibit all variants. Nevertheless, given that HI results correlate with HA hemagglutination and that these epitopes are located in the HA stem, it will be necessary to confirm this using in vitro tests.

Conformational epitopes are more likely to be recognized by antibodies that inhibit hemagglutination. In these proteins, Ellipro predicted a total of 8 conformational epitopes, of which 5 had the highest similarity percentage across H5N2 strains (Figure 3b,c). These five epitopes could be targets for the 30 samples that recognize both strains. One epitope was more similar between H5N1 and H5N2 2006, another was equally similar between H5N2 strains and H5N2 2006 with H5N1. Finally, there was one more epitope shared between H5N1 and H5N2 2024. All of these could be targets for samples that recognize both variants, whether they are H5N1 and H5N2 2006 or H5N1 and H5N2 2024. A crucial consideration is the identification of potential sites recognized as epitopes, as hemagglutination is mediated by the HA1 subunit, particularly within the receptor binding site (RBS). Because of this, there are certain sites with more potential to be related to hemagglutination inhibition by the antibodies in the samples, and most of these are more similar between the two H5N2 strains than with the H5N1 strain; only one epitope is more similar between H5N2 2006 and H5N1 2022.

## 4. Discussion

Some influenza A H5Nx viruses have pandemic potential and high pathogenicity, particularly the H5N1 clade 2.3.4.4b [20,21]. Cross-reactivity of antibodies from humans previously infected or vaccinated against other strains, such as H1N1 or H3N2, could provide protection against H5N1 or H5N2 infection [13,22]. Neutralizing antibodies play a crucial role in the antiviral immune response, and their presence is often associated with high HI titers [23,24,25].

In this study, some samples showed antibodies against more than one viral strain, and positive samples for H5N2 strains correlated more closely with each other than with the H5N1 strain. This can be explained by the similarities between sequences and possible epitopes shared between strains, since H5 proteins’ amino acid sequences are more similar between both H5N2 viruses (91.42%), contrary to H5N1 virus (81.49%). This is expected, since H5N2 viruses have limited pathogenicity, while H5N1 is highly pathogenic, and this difference is reflected in sequence variations in the H5 proteins [26,27]. It has been suggested that HI titers (>1:40) can reduce the infection with H1N1 or H3N2 seasonal influenza viruses, but the same criteria have not been used for other viruses such as H5N1. However, a previous study has proposed that titers greater than 1:40 may indicate protection [25]. According to our results, two samples showed HI titers of 1:64, suggesting cross-protection, probably induced by repeated seasonal influenza infections or vaccination. Further analyses, such as viral neutralization assays, could test this hypothesis.

HI does not account for additional antibody functions, such as Fc-mediated effector mechanisms and neutralization assays, which would be required to confirm these interpretations. Specifically, Fc-mediated effector mechanisms can provide insight into the in vivo effector function of these antibodies, which is crucial to assess for potential in vivo protection [28,29]. Additionally, neutralization assays are not limited to the HA1 region, as the hemagglutination inhibition assay, but can also detect neutralizing antibodies that bind to the HA2 region [30].

According to our results, H5N2 2024 had the highest titers in positive samples, suggesting greater recognition, which can be explained by cross-reactivity with seasonal viruses. This strain was isolated in Texcoco, State of Mexico, and caused an infection in a human during 2024 [8]. Since the samples used in this study were collected in 2022, human exposure to this virus is not considered a cause of seroconversion, supporting cross-reactivity as the most likely reason for the high titers. These findings are relevant to the ongoing spread and increasing mammalian adaptation of the H5N1 clade 2.3.4.4b in North America, emphasizing the need to understand baseline cross-reactive immunity in human populations [31].

The 2006 IAV H5N2 had the highest number of positives after the 2024 H5N2. The 2006 IAV H5N2 has not yet been observed in humans or other mammals, so that the positive results can be attributed to cross-reactivity with seasonal viruses. The serological antibody response against avian influenza viruses is typically not evaluated in humans, since they have not historically posed a major threat to human health [32]. However, our results show differences in cross-reactive responses across strains of avian influenza viruses. H5N1 had the lowest number of positive human serum antibody samples. The presence of antibodies against H5N1 in serum samples from individuals vaccinated against seasonal influenza viruses has been demonstrated, with potential heterosubtypic immunity against H5N1 at low levels, and is supported by our findings [14,25].

This study reveals a tendency for the younger population to have higher levels of serum antibodies against H5N1 than those over 60 years old, consistent with other reports [25]. For all strains, the highest titers of HI were lower in the oldest age group but tended to be higher among younger populations. On the other hand, the percentage of positive samples varied, being higher in the oldest age group for H5N2 strains than for the H5N1 strain. One limitation of this study is the sample size of the elderly population; this should be further evaluated and confirmed among elderly populations. The age-related patterns observed may reflect differences in immune imprinting or vaccine history across birth cohorts, thereby influencing the specificity and magnitude of cross-reactive antibody responses. These results should be confirmed with a larger sample size, comparing individuals with and without seasonal vaccination, and using neutralization assays to corroborate our observations. Additionally, information on vaccination against seasonal influenza viruses would have improved the interpretation of our findings.

## 5. Conclusions

Avian influenza viruses are constantly evolving, and some, like the H5N1 virus, pose a high pandemic risk. Because of this, it is fundamental to continue monitoring the evolution and adaptation of these viruses, as well as to estimate the extent of the human immune response towards them. In our study, we demonstrated that human serum samples from an adult population contain antibodies against the H5N1 strain, as assessed by hemagglutination inhibition. We also observed that the highest HI titers were associated with the IAV H5N2 2024. The proportion of positive samples tended to be higher for the IAV H5N2 2006 and IAV H5N2 2024 in the oldest groups (>50 years old), contrary to H5N1. In contrast, the highest titers were obtained for the younger populations in all cases, indicating a difference in hemagglutination inhibition activity among the adult population, even when antibody levels might be higher with age. Our findings suggest low immune protection against the H5N1 virus and underscores the importance of actions such as seasonal vaccination and ongoing serological surveillance as a crucial component of preparedness for future influenza pandemics.

## Figures and Tables

**Table 1:** Positivity rate of antibodies anti-H5N1 and H5N2 according to age and sex, considering a threshold of 1:32 for the HI test.

Viral Strain	Sex		Age Group				
	Male	Female	18–30	31–40	41–50	51–60	61–76
H5N2 2006	22.4% (48/214)	16% (42/262)	15.2% (12/79)	12.6% (20/159)	16.1% (24/149)	34.2% (27/79)	70% (7/10)
Highest titer	128	128	128	64	128	64	64
H5N2 2024	12.6% (27/214)	9.2% (24/262)	3.8% (3/79)	8.8% (14/159)	13.4% (20/149)	15.2% (12/79)	20% (2/10)
Highest titer	256	128	256	128	256	128	64
H5N1 2022	1.9% (4/214)	3% (8/262)	6.3% (5/79)	1.9% (3/159)	2% (3/149)	1.3% (1/79)	0% (0/10)
Highest titer	64	64	64	64	64	32	16

## Data Availability

The data supporting the findings of this study are available within the article.

## Supplementary Materials

The following supporting information can be downloaded at: https://www.mdpi.com/article/10.3390/pathogens15040352/s1, Supplementary Table S1. Primers and probes are used to detect avian influenza virus genes; Supplementary Table S2. Similarity percentage between linear epitopes predicted for the hemagglutinin of three influenza strains; Supplementary Table S3. Similarity percentage between conformational epitopes predicted for the hemagglutinin of three influenza strains.

## Abbreviations

The following abbreviations are used in this manuscript:

HI	Hemagglutinin inhibition
RBS	Receptor Binding Site
