# Supplementary material for: Antibody Recognition of Highly and Low-Pathogenic A/H5Nx Influenza Viruses in Sera of Mexican Donors
Source: Pathogens. 2026 Mar 26;15(4):352. doi: 10.3390/pathogens15040352 (PMC13118889; doi:10.3390/pathogens15040352)
Supplement: Supplementary file 1 [file pathogens-15-00352-s001.zip › pathogens-4170488-supplementary.pdf]

Antibody Recognition of Highly and Low-Pathogenic A/H5Nx Influenza Viruses in Sera of Mexican Donors

Maritza Cordero-Ortiz [1], Mario Solís-Hernández [2], Marlen Cayetano-Mondragón [2], Nadia Carrillo Guzmán [2],
Olivia Valenzuela [3], Verónica Mata-Haro [4], Luis G. Giménez-Lirola [5] and Jesús Hernández [1,*]

Supplementary materials.

One-Step RT-PCR protocol to detect viral genes

Viral RNA extraction is performed using the nucleic acid extraction kit (B200-32, Zybio) and the EXM 3000 automated system (Zybio), according to the manufacturer's instructions. Detection of the matrix (M) gene was performed by real-time reverse transcription PCR (qRT-PCR) on an Applied Biosystems 7500 platform, using the AgPath-ID™ One-Step RT-PCR kit (Applied Biosystems #4387391) in a final reaction volume of 25 µl. Amplification conditions consisted of reverse transcription at 45°C for 10 minutes, followed by initial denaturation at 95°C for 10 minutes, and 45 cycles of 94°C for 1 second and 60°C for 30 seconds. Primers and probes were diluted to working concentrations of 20 pmol/µL and 6 pmol/µL, respectively, before use.

Supplementary Table S1. Primers and probes are used to detect avian influenza virus genes.

Viral protein	Primer
AIV (Matrix)[a]	M-124 RV: 5′-TGC AAA AAC ATC TTC AAG TCT CTG-3′
AIV (Matrix)[a]	M+25 FW: 5′-AGA TGA GTC TTC TAA CCG AGG TCG-3′
AIV (Matrix)[a]	M+64 Probe: 5′-FAM–TCA GGC CCC CTC AAA GCC GA–BHQ-3′
APMV1 (Matrix)[b]	M+4100 FW 5′-AGT GAT GTG CTC GGA CCT TC-3′
APMV1 (Matrix)[b]	M+4169 5′-[FAM]TTC TCT AGC AGT GGG ACA GCC TGC[TAMRA]-3′
APMV1 (Matrix)[b]	M-4220 RV 5′-CCT GAG GAG AGG CAT TTG CTA-3′
AIV (HA-H5)	RV-H5: 5′-AGA CCA GCT ACC ATG ATT GC-3′
AIV (HA-H5)[c]	H5+1456 FW NA: 5′-ACG TAT GAC TAT CCA CCA TAC TCA-3′
AIV (HA-H5)[c]	H5+1456 FW EA: 5′-ACG TAT GAC TAC CCG CAG TAT TCA-3′
AIV (HA-H5)	H5+1592 FW MEX: 5′-AAT CAA CAG GGA CTT ATC AGA TAC-3′
AIV (HA-H5)[c]	Probe 1637: 5′-FAM–TCA ACA GTG GCG AGT TCC CTA GCA–BHQ1-3′
AIV (HA-H7)[d]	H7 + 1515 FW 5′-ATG GAG AGC ATA AGG AAC-3′
AIV (HA-H7)[d]	H7 - 1628 RV 5′ CCG AAG CTA AAC CAT AAG-3
AIV (HA-H7)[d]	Probe 1585 5′-[6-FAM]-CAGATAGACCCAGTGAAATTGAGT-[BHQ1]-3′

Notes :

[a] World Organisation for Animal Health (WOAH). Avian Influenza (Infection with Avian Influenza Viruses). In Manual of Diagnostic Tests and Vaccines for Terrestrial Animals; WOAH: Paris, France, 2023. Available online: https://www.woah.org/es/que-hacemos/normas/codigos-y-manuales/#chapter/?rid=335&volume_no=3&ismanual=true&language=104&standard_type=6&animal_type=7 (accessed on 3 March 2026).

[b] Spackman E, Suarez DL. Detection and identification of the H5 hemagglutinin subtype by real-time RT-PCR. Methods Mol Biol. 2008;436:27-33. doi: 10.1007/978-1-59745-279-3_5. PMID: 18370038.

[c] Wise MG, Suarez DL, Seal BS, Pedersen JC, Senne DA, King DJ, Kapczynski DR, Spackman E2004.Development of a Real-Time Reverse-Transcription PCR for Detection of Newcastle Disease Virus RNA in Clinical Samples. J Clin Microbiol42:.https://doi.org/10.1128/jcm.42.1.329-338.2005

[d] Spackman E, Ip HS, Suarez DL, Slemons RD, Stallknecht DE. Analytical validation of a real-time reverse transcription polymerase chain reaction test for Pan-American lineage H7 subtype Avian influenza viruses. J Vet Diagn Invest. 2008 Sep;20(5):612-6. doi: 10.1177/104063870802000512. PMID: 18776094.

Supplementary Table S2. Similarity percentage between linear epitopes predicted for the hemagglutinin of three influenza strains.

Epitopes	H5N2 2006 y H5N2 2024	H5N2 2006 y H5N1 2022	H5N2 2024 y H5N1 2022
1	100	75	75
2	96.5	86.2	89.7
3	77.8	77.8	66.7
4	81	56.9	62.1
5	88.2	52.9	64.7
6	76.9	53.8	46.2
7	95.2	80.9	80.9
8	88.9	66.7	55.6
9	91.7	75	83.3
10	78.6	57.1	64.3
11	92.9	95.2	92.9
12	83.3	83.3	66.7
13	100	100	100
14	100	88.9	88.9
15	100	100	100
16	91.7	83.3	83.3

Supplementary Table S3. Similarity percentage between conformational epitopes predicted for the hemagglutinin of three influenza strains.

Epitopes	H5N2 2006 y H5N2 2024	H5N2 2006 y H5N1 2022	H5N2 2024 y H5N1 2022
1	66.7	100	66.7
2	75.9	55.2	58.6
3	90.9	59.1	68.2
4	87.5	75	72.9
5	86.7	66.7	66.7
6	93	93	95.3
7	94.1	94.1	88.2
8	93	87.2	88.4
